# Attention-Deficit/Hyperactivity Disorder Diagnoses in Finland During the COVID-19 Pandemic

**DOI:** 10.1001/jamanetworkopen.2024.18204

**Published:** 2024-06-27

**Authors:** Kirsi Auro, Ida Holopainen, Markus Perola, Aki S. Havulinna, Anu Raevuori

**Affiliations:** 1 Division of Adolescent Psychiatry, Department of Psychiatry, Helsinki University Hospital, Helsinki, Finland; 2 Faculty of Medicine, University of Helsinki, Clinicum, Helsinki, Finland; 3 Department of Public Health and Welfare, Finnish Institute for Health and Welfare-THL, Helsinki, Finland; 4 Institute for Molecular Medicine Finland, FIMM-HiLIFE, University of Helsinki, Helsinki, Finland

## Abstract

**Importance:**

Several reports suggest an increase in attention-deficit/hyperactivity disorder (ADHD) symptoms during the COVID-19 pandemic. This nationwide study assessed new ADHD diagnoses and ADHD prevalence before and during the pandemic.

**Objective:**

To investigate trends in new ADHD diagnoses, prevalence, and ADHD medication use from 2015 to 2022 in Finland.

**Design, Setting, and Participants:**

This longitudinal cohort study comprised the entire Finnish population. ADHD diagnoses and medication use were obtained from nationwide registers and assessed at 3 time points: in 2015 and before (2020) and after (2022) the pandemic. Data were analyzed from January 2015 to June 2022.

**Main outcomes and Measures:**

New ADHD diagnoses, ADHD lifetime prevalence, and ADHD medication use.

**Results:**

The cohort comprised 5 572 420 individuals (2 819 645 women [50.6%]). Lifetime prevalence of ADHD increased by 2.7-fold during 2015 to 2022; prevalence was 1.02% in 2015 (95% CI, 1.01%-1.03%), 1.80% in 2020 (95% CI, 1.79%-1.81%), and 2.76% in 2022 (95% CI, 2.75%-2.77%). Young men aged 13 to 20 years had the highest lifetime prevalence of 11.68% (95% CI, 11.56%-11.81%) in 2022. New ADHD diagnoses doubled during the pandemic, from 238 per 100 000 in 2020 to 477 per 100 000 in 2022. The pandemic-associated incremental increase in new diagnoses was 18.60% (95% CI, 16.47%-20.49%; 9482 per 50 897 cases). Young women aged 13 to 20 years had a 2.6-fold increase in new diagnoses during the pandemic, from 577 per 100 000 in 2020 to 1488 per 100 000 in 2022, and women aged 21 to 30 years had a 3.0-fold increase, from 361 per 100 000 to 1100 per 100 000. New diagnoses increased by 2.9-fold among those older than 55 years (from 5 per 100 000 to 13 per 100 000 in women and from 5 per 100 000 to 14 per 100 000 in men). Boys younger than 13 years had the highest absolute rate of new ADHD diagnoses in 2022 (1745 per 100 000), but boys and young men younger than 21 years did not show a significant incremental increase in new diagnoses. Lifetime prevalence of ADHD medication purchases was 0.57% (95% CI, 0.56%-0.58%) in 2015 (31 771 [55.62%] of those with ADHD diagnosis), 1.15% (95% CI, 1.14%-1.16%) in 2020 (64 034 [63.83%]), and 1.69% (95% CI 1.68%-1.70%) in 2022 (92 557 [61.43%]), respectively.

**Conclusions and Relevance:**

In this nationwide cohort study, new ADHD diagnoses and ADHD prevalence showed significant increase in Finland during the pandemic. ADHD medication use did not increase in relation to ADHD diagnoses. These results highlight potential adverse outcomes of pandemic-associated changes in living conditions.

## Key Points

**Question:**

Did attention-deficit/hyperactivity disorder (ADHD) increase during the COVID-19 pandemic in the Finnish population?

**Findings:**

In this cohort study with more than 5.5 million participants, new ADHD diagnoses doubled during the pandemic between 2020 and 2022 in Finland; a significant increase in ADHD was observed in the population, except among male participants younger than 21 years and among adults older than 55 years. The largest, 3-fold increase was observed in female participants aged 13 to 30 years.

**Meaning:**

These findings suggest new ADHD diagnoses increased significantly in Finland during the pandemic.

## Teaser

This cohort study evaluates trends in new attention-deficit/hyperactivity disorder (ADHD) diagnoses, prevalence, and ADHD medication use before and during the COVID-19 pandemic in Finland.

## Introduction

Attention-deficit/hyperactivity disorder (ADHD) is a common neurodevelopmental disorder characterized by persistent inattention and/or hyperactivity-impulsivity that interfere with functioning or development already at childhood.^1,2^ Individuals with ADHD are often challenged by executive dysfunction, including impairment in organizing, setting schedules, and emotion dysregulation such as lowered tolerance for frustration.^3^ ADHD is associated with a range of other psychiatric disorders across the life span^3,4,5^ and predisposes to marginalization from education and labor market.^6^

Systematic reviews based on general population cohorts before the COVID-19 pandemic report the global prevalence of ADHD in childhood (aged <13 years) ranging between 2% to 10%.^7,8,9,10,11^ Interview-based cohort studies^12^ have reported a higher prevalence approaching 20%. In Finland, a previous cohort study^13^ based on parental interviews estimated a current prevalence of 8.5% and lifetime prevalence of 12.6% among adolescents (aged 16-18 years). Among adult populations, a meta-analysis has reported a global prevalence of 6.8% of “symptomatic adult ADHD” and 2.6% of “persistent adult ADHD” with a strict requirement for childhood onset.^14^

Several studies conducted prior to the COVID-19 pandemic suggest a significant increase in ADHD prevalence during the last decades. ADHD has traditionally been considered a condition of childhood and adolescent boys. During the past 10 years, there has been a greater focus on women and adults, and ADHD is reported to be underdiagnosed, especially in girls, older children, and adults.^6,7,15,16^ A Canadian study on primary care units reported a prevalence increase from 6.9% to 8.5% among those younger than 18 years and from 5.7% to 7.3% among those aged 18 to 35 years between 2008 and 2015.^17^ A US population-based survey among 200 000 adolescents reported a prevalence increase from 6.1% to 10.2% between 1997 and 1998 and 2015 and 2016,^18^ and a German nationwide study^19^ based on health insurance funds reported an increase from 5% to 6% between 2009 and 2014 among those younger than 18 years. The adverse effects of the COVID-19 pandemic and related restrictive measures on mental health have been documented worldwide,^20,21^ with populations experiencing more severe pandemic-related stress being at higher risk of mental disorders.^22^ Individuals diagnosed with ADHD before COVID-19 experienced symptom deterioration and increased depressive symptoms.^23,24,25,26,27^ In the US, incident ADHD medication prescriptions increased sharply and more than other behavioral health medications during 2018 and 2022, especially among young adults and women.^28^ A rapid increase in ADHD-like symptoms among previously healthy individuals during the pandemic has been reported.^29,30,31^ Attention disorder, ie, inattention and ADHD-like symptoms, has also been identified as one of the symptoms of post–COVID-19 condition.^32^

Several reports have suggested an increase in ADHD symptoms during the COVID-19 pandemic. This study aimed to investigate the changes in new ADHD diagnoses, ADHD prevalence, and ADHD medication use in the Finnish population between 2015 and 2022 using nationwide registers.

## Methods

We assessed new ADHD diagnoses, ADHD prevalence, and ADHD medication use using nationwide health register data in a population-based cohort study setting. The study has ethical permission from the Finnish National Institute for Health and Welfare. Informed participant consent is not required for register-based studies using anonymized data. This study followed Strengthening the Reporting of Observational Studies in Epidemiology (STROBE) reporting guidelines.

### Study Population

The study population comprised all individuals living in Finland and holding a Finnish social security number between January 1, 2015, and December 31, 2020. ADHD diagnoses from January 1969 to June 2022 were obtained from the National Care Register for Health Care. This register covers nationwide hospital inpatient diagnoses since 1969, hospital outpatient clinic visits since 1998, all public primary care units since 2011, and occupational health care units since 2020. Medication data were gathered from January 1, 1994, to June 30, 2022, from the National Medicine Purchase Register, covering all prescriptions nationwide.

Data from different registers were combined using social security numbers. ADHD diagnoses were identified using the *International Classification of Diseases, Eighth, Ninth *and *Tenth Revisions (ICD-8; ICD-9; ICD-10); ICD-8*, 1969-1986, codes 308.99, 308.3, and 309; *ICD-9,* 1987-1996, code 3140; and *ICD-10,* 1996-present, codes F90 or F98.8 (used for inattentive subtype of ADHD in Finland).^33^ Data on ADHD medication were categorized using the Anatomical Therapeutic Chemical classification,^34^ including methylphenidate (N06BA04), atomoxetine (N06BA09), dexamphetamine (N06BA02), and lisdexamphetamine (N06BA12).

### Outcomes

The main study outcome was the number of new ADHD diagnoses, defined either as the first *ICD*-based primary or secondary ADHD diagnosis or first ADHD medication purchase in Finland between 2015 and 2022. Nationwide pandemic-related restrictions were enforced in Finland during March to June 2020 and lifted during summer 2020. During this first pandemic wave, all education facilities and most of the working life venues were closed. During the second pandemic wave in November 2020 to April 2021, elementary schools stayed open. Reflecting the pandemic-related restrictions, we estimated the number of new ADHD diagnoses per 100 000 at 3 different time periods: (1) during the year 2015 (from the beginning of nationwide data collection), (2) immediately before the pandemic in April 2019 to March 2020, and (3) during the pandemic in July 2021 to June 2022. The proportional rate of new ADHD diagnoses was estimated with half-year intervals from January 2015 to June 2022. To ensure capturing the first diagnosis, we tracked each individual back to birth or the year 1969 (Care Register initiation). Possible trend changes in new ADHD diagnoses before and during the pandemic were analyzed with age- and sex-specific models.

The secondary outcomes were (1) ADHD lifetime prevalence, defined as prevalent ADHD diagnosis or prevalent medication purchase, and (2) lifetime prevalence and period prevalence for ADHD medication use, describing the proportion of the population using ADHD medication at the time. Lifetime prevalence of ADHD and ADHD medication use were also estimated at 3 different time points with half-year intervals, similar to new ADHD diagnoses.

### Statistical Analysis

All analyses were grouped and performed independently by sex and age, including age groups of 0 to 12 years, 13 to 20 years, 21 to 30 years, 31 to 55 years, and older than 55 years. The significance level was set to .05. New ADHD diagnoses in each group were modeled with multivariable Poisson or negative binomial regression. These models were further used in the estimation of excessive new ADHD diagnoses during pandemic. R software version 4.1.3 (R Project for Statistical Computing) was used for statistical analyses (with packages data.table, dplyr, MASS, ggplot2, patchwork, ggsci, scales, and stringr). The eMethods in Supplement 1 describes the statistical analyses in detail. Data were analyzed from January 2015 to June 2022.

## Results

The nationwide study cohort comprised 5 572 420 Finland residents. In 2022, the mean (SD) age was 44.1 (23.6) years; x were women (50.65%), and there were 229 753 086 observed person-years during follow-up (eTable 1 in Supplement 2).

### Outcomes

#### New ADHD Diagnoses by Age and Sex

**Figure 1.**
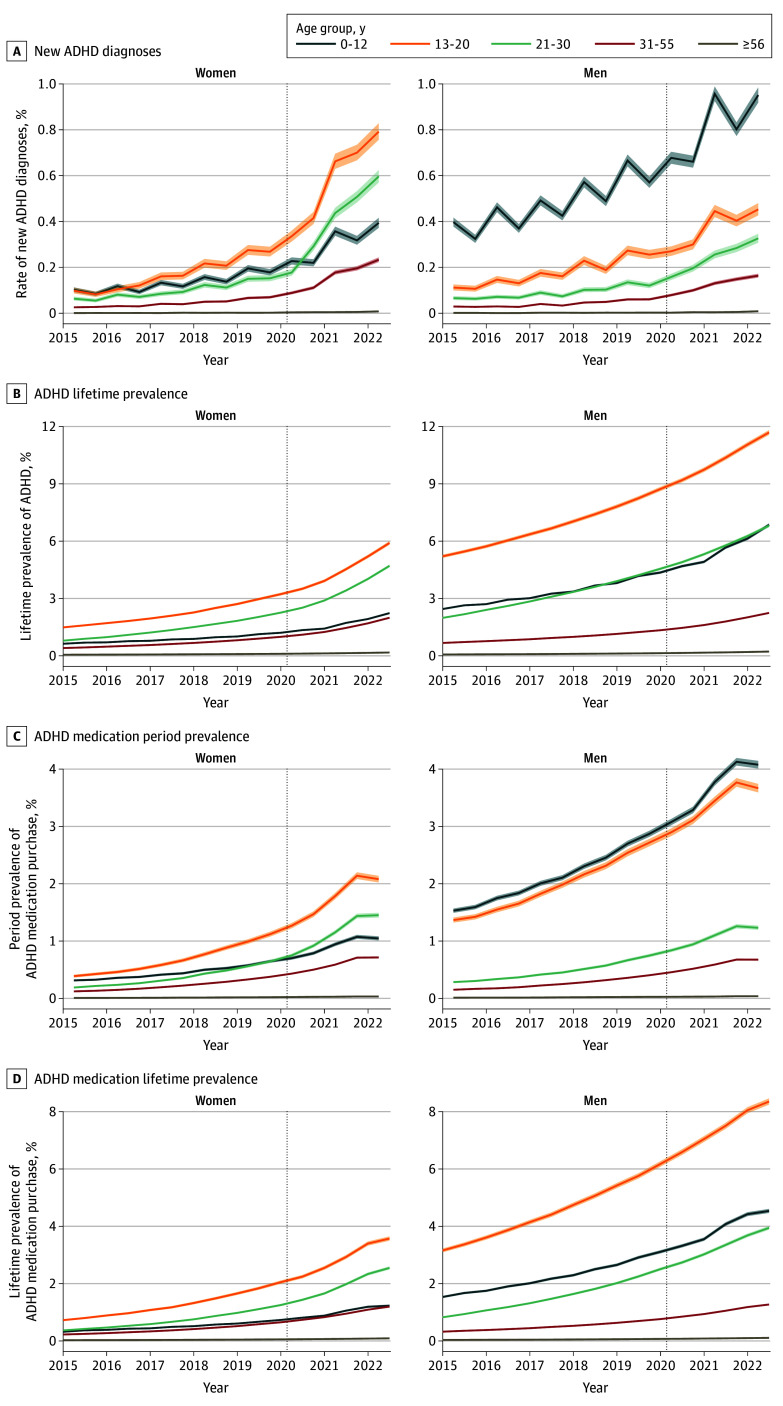
New Diagnoses and Lifetime Prevalence Rates of Attention-Deficit/Hyperactivity Disorder (ADHD) and Period Prevalence and Lifetime Prevalence Rates of ADHD Medication Purchases in the Finnish Population by Age and Sex Between January 2015 and June 2022 Rates of new diagnoses and period prevalence were computed with half-year intervals, and lifetime prevalence was estimated every half year. The population at risk was estimated with midpoint population for new diagnosis rate and period prevalence. Shaded area around the estimated rate represents the Clopper-Pearson 95% CI. The dashed vertical line marks the beginning of the pandemic in Finland (April 1, 2020).

New ADHD diagnoses doubled from 2015 to prepandemic year 2019 to 2020 in Finland, and again doubled from 2019 to 2020 to the pandemic year 2021 to 2022. In 2015, the rate of new ADHD diagnoses per 100 000 was 117 (95% CI, 114-120), in 2019 to 2020, it was 238 per 100 000 (95% CI, 234-242), and in 2022, it was 477 per 100 000 (95% CI, 471-483) (Table 1 and Figure 1A; eFigure 1 in Supplement 1 and eTable 2 in Supplement 2). The rate of new ADHD diagnoses by age and sex was the highest among boys younger than 13 years throughout the study. In 2015, it was 721 per 100 000 (95% CI, 695-747), in 2019 to 2020, it was 1243 per 100 000 (95% CI, 1208-1278), and in 2022, it was 1745 per 100 000 (95% CI, 1701-1789) (Table 1 and Figure 1B; eFigure 1 and eFigure 2 in Supplement 1).

**Table 1.** New Diagnoses (Incidence) of Attention-Deficit/Hyperactivity Disorder (ADHD) and Period Prevalence of ADHD Medication Purchase Measured at 3 Time Periods

Sex, age group, and period	Population proportion		Incident ADHD, No.	ADHD incidence per 100 000 (95% CI)	Prevalent ADHD medication purchase, No.	ADHD medication purchase period prevalence per 100 000 (95% CI)
	No. (95% CI)	% (95% CI)				
Girls and young women						
0-12 y						
January 1-December 31, 2015	388 596 (387 375-389 818)	6.97 (6.95-6.99)	725	186 (173-200)	1474	379 (360-398)
April 1, 2020-March 31, 2019	368 493 (367 304-369 683)	6.62 (6.60-6.64)	1431	388 (368-408)	2869	778 (750-807)
July 1, 2022-June 30, 2022	328 682 (327 559-329 806)	5.98 (5.96-6.00)	2327	707 (679-736)	4011	1220 (1183-1258)
13-20 y						
January 1-December 31, 2015	240 750 (239 789-241 712)	4.32 (4.30-4.33)	436	181 (164-198)	1158	481 (453-509)
April 1, 2019-March 31, 2020	233 407 (232 461-234 354)	4.19 (4.18-4.21)	1348	577 (547-608)	3235	1385 (1338-1433)
July 1, 2022-June 30, 2022	236 459 (235 506-237 413)	4.30 (4.29-4.32)	3520	1488 (1440-1537)	5946	2514 (2451-2578)
Boys and young men						
0-12 y						
January 1-December 31, 2015	406 821 (405 571-408 072)	7.29 (7.27-7.32)	2934	721 (695-747)	7417	1823 (1782-1864)
April 1, 2019-March 31, 2020	385 746 (384 529-386 964)	6.93 (6.91-6.95)	4797	1243 (1208-1278)	13 201	3422 (3365-3479)
July 1, 2022-June 30, 2022	343 942 (342 793-345 092)	6.26 (6.24-6.28)	6003	1745 (1701-1789)	15 829	4602 (4532-4672)
13-20 y						
January 1-December 31, 2015	257 344 (256 350-258 339)	4.61 (4.60-4.63)	563	218 (201-237)	4028	1565 (1517-1613)
April 1, 2019-March 31, 2020	246 509 (245 536-247 483)	4.43 (4.41-4.45)	1287	522 (494-550)	7618	3090 (3022-3159)
July 1, 2022-June 30, 2022	247 650 (246 675-248 626)	4.51 (4.49-4.52)	2120	856 (820-892)	10 347	4178 (4099-4257)
Women						
21-30 y						
January 1-December 31, 2015	347 136 (345 982-348 291)	6.22 (6.20-6.24)	411	118 (107-130)	864	248 (232-265)
April 1, 2019-March 31, 2020	333 621 (332 489-334 754)	5.99 (5.97-6.01)	1055	316 (297-335)	2614	783 (753-813)
July 1, 2022-June 30, 2022	319 596 (318 488-320 704)	5.81 (5.79-5.83)	3516	1100 (1064-1136)	5471	1711 (1667-1757)
31-55 y						
January 1-December 31, 2015	873 588 (871 757-875 420)	15.66 (15.63-15.69)	463	53 (48-57)	1309	149 (141-158)
April 1, 2019-March 31, 2020	856 789 (854 975-858 604)	15.39 (15.36-15.42)	1206	140 (132-148)	3792	442 (428-456)
July 1, 2022-June 30, 2022	850 790 (848 983-852 598)	15.48 (15.45-15.51)	3639	427 (413-441)	7013	824 (805-843)
≥56 y						
January 1-December, 31 2015	974 729 (972 794-976 665)	17.48 (17.44-17.51)	19	1.95 (1.17-2.92)	82	8.41 (6.69-10.33)
April 1, 2019-March 31, 2020	1 024 706 (1 022 722-1 026 691)	18.41 (18.38-18.44)	49	4.78 (3.54-6.21)	226	22 (19-25)
July 1, 2022-June 30, 2022	1 046 316 (1 044 312-1 048 321)	19.04 (19.00-19.07)	143	13 (11-16)	382	36 (32-40)
All age groups						
January 1-December 31, 2015	2 824 799 (2 821 505-2 828 094)	50.65 (50.60-50.69)	2054	72 (69-75)	4887	173 (168-177)
April 1, 2019-March 31, 2020	2 817 016 (2 813 727-2 820 306)	50.60 (50.56-50.65)	5089	180 (175-185)	12 736	452 (444-459)
July 1, 2022-June 30, 2022	2 781 843 (2 778 574-2 785 112)	50.61 (50.57-50.65)	13 145	472 (464-480)	22 823	820 (809-831)
Men						
21-30						
January 1-December 31, 2015	371 286 (370 092-372 481)	6.66 (6.64-6.68)	478	128 (117-140)	1335	359 (340-379)
April 1, 2019-March 31, 2020	355 703 (354 535-356 872)	6.39 (6.37-6.41)	911	256 (239-272)	3148	885 (854-916)
July 1, 2022-June 30, 2022	340 978 (339 834-342 123)	6.20 (6.18-6.22)	2077	609 (583-635)	5011	1469 (1429-1510)
31-55						
January 1-December 31, 2015	905 452 (903 587-907 317)	16.23 (16.20-16.26)	515	56 (52-61)	1656	182 (174-191)
April 1, 2019-March 31, 2020	895 186 (893 332-897 041)	16.08 (16.05-16.11)	1129	126 (118-133)	4123	460 (446-474)
July 1, 2022-June 30, 2022	891 860 (890 010-893 711)	16.23 (16.20-16.26)	2774	311 (299-322)	6839	766 (748-785)
≥56 y						
January 1-December 31, 2015	811 845 (810 079-813 611)	14.56 (14.53-14.58)	23	2.83 (1.80-4.10)	111	13 (11-16)
April 1, 2019-March 31, 2020	866 640 (864 816-868 465)	15.57 (15.54-15.60)	47	5.42 (3.98-7.08)	245	28 (24-31)
July 1, 2022-June 30, 2022	890 266 (888 417-892 116)	16.20 (16.17-16.23)	126	14 (11-16)	365	41 (36-45)
All age groups						
January 1-December 31 2015	2 752 748 (2 749 497-2 756 000)	49.35 (49.31-49.40)	4513	163 (159-168)	14 547	528 (519-537)
April 1, 2019-March 31, 2020	2 749 784 (2 746 534-2 753 035)	49.40 (49.35-49.44)	8171	297 (290-303)	28 335	1030 (1018-1042)
July 1, 2022-June 30, 2022	2 714 696 (2 711 467-2 717 926)	49.39 (49.35-49.43)	13 100	482 (474-490)	38 391	1414 (1400-1428)
All age and sex groups						
January 1-December 31, 2015	5 577 547 (5 572 919-5 582 176)	100.00 (100.00-100.00)	6567	117 (114-120)	19 434	348 (343-353)
April 1, 2019-March 31, 2020	5 566 800 (5 562 176-5 571 425)	100.00 (100.00-100.00)	13 260	238 (234-242)	41 071	737 (730-744)
July 1, 2022-June 30, 2022	5 496 539 (5 491 944-5 501 135)	100.00 (100.00-100.00)	26 245	477 (471-483)	61214	1113 (1104-1122)

**Figure 2.**
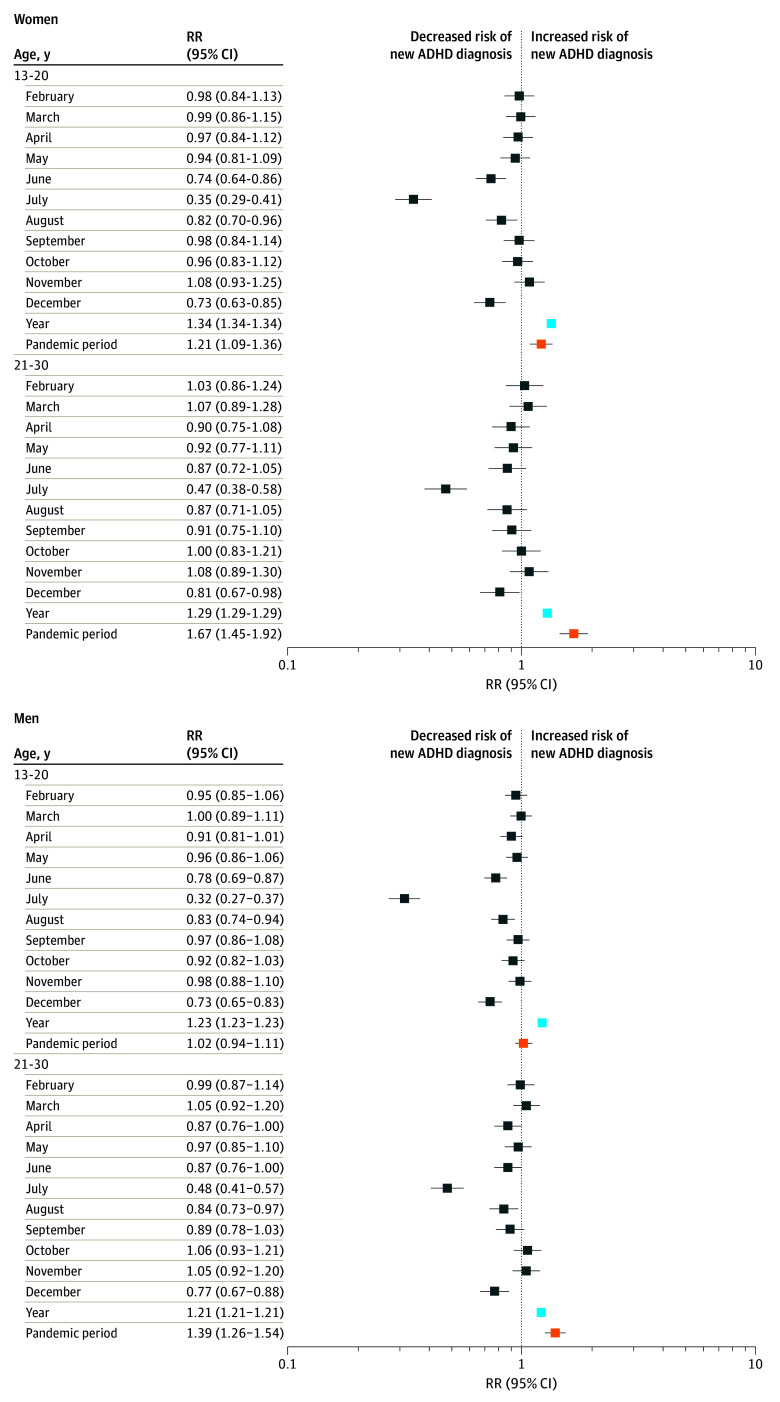
Forest Plot of Estimated New Attention-Deficit/Hyperactivity Disorder (ADHD) Diagnosis Rate Ratios (RRs) Before and After the Pandemic Period RRs and their 95% CIs were obtained from the negative binomial regression that was modeled separately for women and men in specified age groups. The models were adjusted for overall increasing trend that was modeled with continuous date count, here rescaled to represent yearly effect (light blue); seasonal effect as categorical variable for each month (dark blue), in which January was used as baseline; and possible effect of the COVID-19 pandemic with dummy variable (orange). The squares represent the estimated RR that is the exponential transformation of the covariate estimate and horizontal bars represent their 95% CIs, and they are colored according to model variables. The RR of the pandemic period (orange) describes the ratio of new ADHD diagnosis rates during vs before pandemic in each model.

The most significant increase in new ADHD diagnoses was seen among girls and young women aged 13 to 20 years (2.6-fold compared with prepandemic year, from 577 per 100 000 in 2020 to 1488 per 100 000 in 2022) and 21 to 30 years 2.8-fold compared with prepandemic year) (Figure 2). Women aged 21 to 30 years had a 3.0-fold increase, from 361 per 100 000 to 1100 per 100 000. Among those older than 55 years, the rate of new ADHD diagnoses also increased 2.9-fold in both sexes (from 5 per 100 000 to 13 per 100 000 in women and from 5 per 100 000 to 14 per 100 000 in men), but the increase was not statistically significant in exceeding the prepandemic growth trend (Figure 2). Next, we estimated the number of incremental new ADHD diagnoses during the pandemic over the overall increasing trend using fitted models. The total number of incremental cases was 9482 (95% CI, 8396-10 551 [67.4% women]) during the pandemic, representing 18.60% of all new ADHD diagnoses (95% CI, 16.47%-20.49%) (Table 2).

**Table 2.** Additional New Diagnoses of Attention-Deficit/Hyperactivity Disorder (ADHD) During COVID-19 Pandemic in Finland

Sex and age group	Observed, No.	No. (95% CI)			Proportion of additional cases from observed, % (95% CI)
		Model estimate	Model estimate without pandemic effect	Estimated additional cases	
Girls and young women					
0-12 y	4781	4766 (4592 to 4951)	4050 (3890 to 4211)	716 (476 to 959)	14.98 (9.96 to 20.06)
13-20 y	6426	6387 (6072 to 6704)	5261 (4990 to 5529)	1125 (709 to 1547)	17.52 (11.03 to 24.07)
Boys and young men					
0-12 y	13 128	13 110 (12 689 to 13 530)	12 792 (12 371 to 13 212)	317 (−279 to 907)	2.42 (−2.13 to 6.91)
13-20 y	4281	4281 (4134 to 4439)	4192 (4044 to 4348)	89 (−126 to 305)	2.08 (−2.94 to 7.12)
Women					
21-30 y	6199	6087 (5706 to 6483)	3639 (3402 to 3885)	2447 (1991 to 2909)	39.49 (32.12 to 46.93)
31-55 y	6525	6405 (6072 to 6738)	4378 (4143 to 4618)	2026 (1605 to 2432)	31.06 (24.60 to 37.27)
≥56	266	266 (234 to 298)	194 (167 to 222)	71 (30 to 114)	27.03 (11.28 to 42.86)
All age groups	24 197	23 913 (23 270 to 24 524)	17 525 (17 063 to 17 990)	6388 (5604 to 7170)	26.40 (23.16 to 29.63)
Men					
21-30 y	3948	3932 (3765 to 4100)	2822 (2692 to 2952)	1109 (900 to 1321)	28.11 (22.80 to 33.46)
31-55 y	5215	5182 (4965 to 5404)	3646 (3478 to 3812)	1536 (1261 to 1815)	29.46 (24.18 to 34.80)
≥56	218	218 (190 to 247)	176 (151 to 203)	41 (2 to 81)	19.00 (0.92 to 37.16)
All age groups	26 790	26 724 (26 195 to 27 254)	23 630 (23 134 to 24 116)	3093 (2356 to 3814)	11.55 (8.79 to 14.24)
All age and sex groups	50 987	50 638 (49 810 to 51 449)	41 156 (40 473 to 41 836)	9482 (8396 to 10 551)	18.60 (16.47 to 20.69)

Occupational health care joined the Care Register in 2020, potentially contributing to the rate of new ADHD diagnoses. To assess its potential outcomes, we conducted sensitivity analyses excluding the occupational health care cases (eTable 3, eTable 4, eTable 5, eTable 6, eFigure 3 and eFigure 4 in Supplement 1). Although overlapping with the pandemic period, the inclusion of occupational health registry cases did not explain the observed incremental new ADHD diagnoses.

#### ADHD Prevalence by Age and Sex

Next, we assessed the outcomes of the pandemic period on ADHD prevalence. We calculated lifetime prevalence during 3 different time points, similar to new ADHD diagnoses (Table 3). Lifetime prevalence of ADHD in the whole population in 2015 was 1.02% (56 790 diagnoses; 95% CI, 1.01%-1.03%), the prepandemic lifetime prevalence in 2020 was 1.80% (100 228 diagnoses; 95% CI, 1.79%-1.81%) and after the pandemic in June 2022, it was 2.76% (150 771 diagnoses; 95% CI, 2.75%-2.77%). Between 2015 to 2020 the ADHD case number in Finland increased by 43 181, whereas during the pandemic between April 2020 and June 2022, a total of 50 028 new diagnoses were observed (Table 3). Boys and young men aged 13 to 20 years had the highest lifetime ADHD prevalence throughout the study (5.72% in 2015, 8.95% in 2020, and 11.68% [95% CI, 11.56%-11.81%] in 2022) (Table 3; eFigure 5 in Supplement 1). Inclusion of occupational health care cases did not significantly change the prevalence (eTable 7 in Supplement 2). The male to female ratio for the lifetime ADHD prevalence in 2022 was lower, 1.8:1. We observed the largest discrepancy in male to female ratio of 2.7:1 among those aged 0 to 12 years, whereas among those aged 13 to 20 years, the male to female ratio was 2.1:1. The male to female ratio for the lifetime prevalence of medication use was 2:1 for the whole population.

**Table 3.** Lifetime Prevalence of Attention-Deficit/Hyperactivity Disorder (ADHD) and ADHD Medication Purchase Measured at 3 Time Points

Date	Population proportion		Prevalent ADHD, No.	ADHD lifetime prevalence, % (95% CI)	Prevalent ADHD medication purchase, No.	ADHD medication purchase lifetime prevalence, % (95% CI)
	No.	% (95% CI)				
Women						
0-12 y						
December 31, 2015	388 189	6.96 (6.94-6.98)	2717	0.70 (0.67-0.73)	1487	0.38 (0.36-0.40)
March 31, 2020	364 674	6.56 (6.54-6.58)	4652	1.28 (1.24-1.31)	2846	0.78 (0.75-0.81)
June 30, 2022	313 091	5.73 (5.71-5.75)	6977	2.23 (2.18-2.28)	3842	1.23 (1.19-1.27)
13-20 y						
December 31, 2015	238 518	4.27 (4.26-4.29)	4065	1.70 (1.65-1.76)	2110	0.88 (0.85-0.92)
March 31, 2020	234 077	4.21 (4.19-4.23)	7913	3.38 (3.31-3.45)	5072	2.17 (2.11-2.23)
June 30, 2022	237 245	4.34 (4.32-4.36)	14 004	5.90 (5.81-6.00)	8472	3.57 (3.50-3.65)
21-30 y						
December 31, 2015	346 026	6.20 (6.18-6.22)	3365	0.97 (0.94-1.01)	1608	0.46 (0.44-0.49)
March 31, 2020	330 741	5.95 (5.93-5.97)	7876	2.38 (2.33-2.43)	4418	1.34 (1.30-1.38)
June 30, 2022	316 181	5.79 (5.77-5.81)	14 868	4.70 (4.63-4.78)	8051	2.55 (2.49-2.60)
31-55 y						
December 31, 2015	872 759	15.64 (15.61-15.67)	4146	0.48 (0.46-0.49)	2353	0.27 (0.26-0.28)
March 31, 2020	855 030	15.37 (15.34-15.40)	8888	1.04 (1.02-1.06)	5878	0.69 (0.67-0.71)
June 30, 2022	849 393	15.54 (15.51-15.57)	16 871	1.99 (1.96-2.02)	10 142	1.19 (1.17-1.22)
≥56						
December 31, 2015	980 521	17.57 (17.54-17.60)	569	0.06 (0.05-0.06)	268	0.03 (0.02-0.03)
March 31, 2020	1 029 621	18.51 (18.48-18.55)	1073	0.10 (0.10-0.11)	569	0.06 (0.05-0.06)
June 30, 2022	1 050 083	19.22 (19.18-19.25)	1763	0.17 (0.16-0.18)	920	0.09 (0.08-0.09)
All age groups						
December 31, 2015	2 826 013	50.64 (50.60-50.68)	14 862	0.53 (0.52-0.53)	7826	0.28 (0.27-0.28)
March 31, 2020	2 814 143	50.60 (50.56-50.64)	30 402	1.08 (1.07-1.09)	18 783	0.67 (0.66-0.68)
June 30, 2022	2 765 993	50.61 (50.57-50.66)	54 483	1.97 (1.95-1.99)	31 427	1.14 (1.12-1.15)
Men						
0-12 y						
December 31, 2015	406 096	7.28 (7.26-7.30)	10 964	2.70 (2.65-2.75)	7092	1.75 (1.71-1.79)
March 31, 2020	381 626	6.86 (6.84-6.88)	17 476	4.58 (4.51-4.65)	12 530	3.28 (3.23-3.34)
June 30, 2022	327 580	5.99 (5.97-6.01)	22 432	6.85 (6.76-6.93)	14 857	4.54 (4.46-4.61)
13-20 y						
December 31, 2015	254 909	4.57 (4.55-4.59)	14 592	5.72 (5.63-5.81)	9187	3.60 (3.53-3.68)
March 31, 2020	246 826	4.44 (4.42-4.46)	22 089	8.95 (8.84-9.06)	15 752	6.38 (6.29-6.48)
June 30, 2022	248 349	4.54 (4.53-4.56)	29 014	11.68 (11.56-11.81)	20 740	8.35 (8.24-8.46)
21-30 y						
December 31, 2015	370 222	6.63 (6.61-6.65)	8871	2.40 (2.35-2.45)	3935	1.06 (1.03-1.10)
March 31, 2020	352 757	6.34 (6.32-6.36)	16 635	4.72 (4.65-4.79)	9211	2.61 (2.56-2.66)
June 30, 2022	337 263	6.17 (6.15-6.19)	22 953	6.81 (6.72-6.89)	13 296	3.94 (3.88-4.01)
31-55 y						
December 31, 2015	905 012	16.22 (16.19-16.25)	6893	0.76 (0.74-0.78)	3421	0.38 (0.37-0.39)
March 31, 2020	893 665	16.07 (16.04-16.10)	12 419	1.39 (1.37-1.41)	7143	0.80 (0.78-0.82)
June 30, 2022	891 065	16.31 (16.27-16.34)	19 945	2.24 (2.21-2.27)	11 301	1.27 (1.25-1.29)
≥56						
December 31, 2015	818 300	14.66 (14.63-14.69)	608	0.07 (0.07-0.08)	310	0.04 (0.03-0.04)
March 31, 2020	872 204	15.68 (15.65-15.71)	1207	0.14 (0.13-0.15)	615	0.07 (0.07-0.08)
June 30, 2022	894 600	16.37 (16.34-16.40)	1944	0.22 (0.21-0.23)	936	0.10 (0.10-0.11)
All age groups						
December 31, 2015	2 754 539	49.36 (49.32-49.40)	41 928	1.52 (1.51-1.54)	23 945	0.87 (0.86-0.88)
March 31, 2020	2 747 078	49.40 (49.36-49.44)	69 826	2.54 (2.52-2.56)	45 251	1.65 (1.63-1.66)
June 30, 2022	2 698 857	49.39 (49.34-49.43)	96 288	3.57 (3.55-3.59)	61 130	2.27 (2.25-2.28)
All age and sex groups						
December 31, 2015	5 580 552	100.00 (100.00-100.00)	56 790	1.02 (1.01-1.03)	31 771	0.57 (0.56-0.58)
March 31, 2020	5 561 221	100.00 (100.00-100.00)	100 228	1.80 (1.79-1.81)	64 034	1.15 (1.14-1.16)
June 30, 2022	5 464 850	100.00 (100.00-100.00)	150 771	2.76 (2.75-2.77)	92 557	1.69 (1.68-1.70)

#### The Lifetime Prevalence and Period Prevalence of ADHD Medication Use

The lifetime prevalence of ADHD medication use in 2015 in the population was 0.57% (95% CI, 0.56%-0.58%; 31 771 [55.62%] of those with prevalent ADHD) and 1.15% in March 2020 (95% CI, 1.14%-1.16%; 64 034 [63.83%] of those with prevalent ADHD) (Table 3 and Figure 1C-D). In June 2022, the lifetime prevalence was 1.69% (95% CI, 1.68%-1.70%; 92 557 [61.43%] of those with prevalent ADHD) (Table 3 and Figure 1C-D). We observed a lifetime prevalence of 0.17% among women and 0.22% among men aged older than 55 years after the pandemic in 2022. While ADHD medication use increased 2.1-fold between 2015 and 2020, the increase was 1.4-fold during the pandemic between 2020 and 2022. Medication use was most common among boys younger than 13 years (14 857 [66.23%] of those with ADHD diagnoses in 2022) and the least common among men older than 55 years (936 [48.15%] of those with ADHD diagnoses in 2022) (Table 3 and Figure 1C-D). We did not observe an incremental increase in ADHD medication use exceeding the increase in new ADHD diagnoses during the pandemic. Inclusion of occupational health care cases did not change the prevalence of medication use (eTable 7 in Supplement 2).

## Discussion

This is, to our knowledge, the first nationwide study assessing new ADHD diagnoses and ADHD prevalence during the COVID-19 pandemic. With the entire Finnish population as a study cohort, we found a rapid, 2-fold increase in new ADHD diagnoses and 1.5-fold increase in ADHD prevalence during the COVID-19 pandemic compared with the immediate prepandemic time. Our findings suggest an overall incremental increase of 18.60% in new ADHD diagnoses during the pandemic; two-thirds of these excessive, pandemic-associated new cases were observed in women. Consequently, the highest increase in new ADHD diagnoses was observed in girls and young women (aged 13-30 years), whose new diagnoses increased 3-fold from the prepandemic year 2019 to 2020 to the pandemic year 2021 to 2022. Among the population older than 55 years, new ADHD diagnoses likewise almost tripled in both men and women, although the absolute numbers of cases were still low. Throughout the study from 2015 to 2022, the highest absolute rates of new ADHD diagnoses were observed among boys younger than 13 years, and the highest lifetime ADHD prevalence rates were among boys and young men aged 13 to 20 years. These young male cohorts were also the only ones not showing pandemic-associated incremental increases in new ADHD diagnoses, potentially stemming from relatively high rates already prepandemic.

We observed a lifetime ADHD prevalence of 2.76% in the entire population, which is in line with global reports.^7,8,9,10,11,14,17,19^ As expected, community-based studies, including cases that were undetected in health care, have reported higher ADHD prevalence rates^12,18^ compared with studies based on cases diagnosed in health care services.^17,19^ Overall, we did not observe an incremental increase in ADHD medication use in relation to increase in ADHD diagnoses (ie, ADHD medication use increased linearly with new diagnoses).

In 2007, Smalley et al^13^ reported the prevalence of definite *Diagnostic and Statistical Manual of Mental Disorders* (Fourth Edition)–defined ADHD among adolescents from the general population to be 8.5% in a birth cohort from Northern Finland. Retrospective childhood ADHD prevalence was 12.6%, and broad lifetime ADHD prevalence 18.2%.^13^ This study was based on parental interviews, and the figures suggest a high number of undiagnosed cases, now potentially surfacing in Finland with pandemic-induced challenges in living conditions.

Smalley et al^13^ estimated a male to female ratio of 3.2:1 for lifetime prevalence. In our study, the male to female ratio for the lifetime ADHD prevalence in 2022 was lower, 1.8:1. We observed the largest discrepancy in male to female ratio of 2.7:1 among those aged 0 to 12 years, whereas among those aged 13 to 20 years, the male to female ratio was 2.1:1, also lower than reported by Smalley et al.^13^ These differences may reflect the use of the entire population as a study cohort, as well as the use of register-based data. We also observed a significant incremental increase in ADHD period prevalence among young women, which may further explain a lower male to female ratio. The prevalence rates and sex distributions observed in this study are largely in line with previous reports on the prevalence rate and male to female ratio of ADHD.^34,35,36^ The male to female ratio for the lifetime prevalence of medication use was 2:1 for the whole population. We did not observe differences in medication use among men and women with ADHD.

During the past decade, ADHD diagnostics in Finland have shifted largely from specialized health care to schools and primary health care units. School environments may create a tendency to surface hyperactivity- and impulsivity-related symptoms more easily, whereas inattentive symptoms may be undetected. This may partly explain a potential accumulation of undiagnosed cases among girls with inattentive symptoms. Pandemic-induced life changes may have induced these underlying cases to surface among girls and young women. Data on ADHD prevalence among older adults are limited, but recent meta-analyses have described lifetime prevalence of 0.23% among those older than 50 years^37^ and 0.8%^14^ and 1.1%^38^ among those older than 60 years. Here we observed a lifetime prevalence of 0.17% among women and 0.22% among men aged older than 55 years after the pandemic in 2022. Although the number of new ADHD cases in this age group increased by 2.7-fold during the pandemic, the lifetime prevalence in Finland among those older than 55 years is still low compared with global meta-analyses.^14,37,38^ This may relate to past clinical conventions and potential underdiagnosis among the older age groups.

Changes in social, occupational, and learning environments, such as loss of routines, lack of face-to-face contacts outside of home, increased demand for online activity, excessive screen time and digital media use, and reduced physical exercise, have appeared to contribute to the increase in ADHD symptoms in other populations.^38,39,40,41,42^ Underlying influences for the observed increase in ADHD symptoms during the pandemic are, however, poorly understood. Classified as a developmental disorder with a childhood onset, the emergence of ADHD in later adolescence or adulthood has been debated.^3,6,43,44^ Twin studies^45,46^ suggest a substantial genetic risk component for ADHD with heritability estimates ranging between 77% and 88% and remaining stable throughout the life span. Established environmental risk factors include early life experiences such as maternal substance abuse during pregnancy, ischemic birth conditions, psychosocial deprivation, and emotional trauma.^43^ In accordance with these findings, adverse living conditions later in life, such as a pandemic lockdown, should have a limited association with new-onset ADHD in older adolescents and adults. On the other hand, despite comparable ADHD symptoms and impairment, individuals with late-onset ADHD have been reported to show fewer behavioral problems in childhood and higher cognitive capacity compared with those with childhood onset.^39^ Pandemic lockdown imposed a sudden increase to attention and executive behavioral demands, coupled with a lack of daily structures and reduced possibilities for physical exercise. These challenges in living conditions may have surfaced ADHD symptoms in individuals previously coping sufficiently in their daily life.

Several studies have reported a connection between increased screen time and inattentive symptoms.^23,28,40,42^ Restrictive measures due to the pandemic pushed people to remote studies or work, exposing many to the jungle of digital platforms, abundant screen time, and increased demands for concentration and self-direction. Closure of after-school activities such as sports during the pandemic induced an increased time spent on computers and other digital gaming platforms. Under these circumstances, underlying challenges with attention and organized behavior may have surfaced, and ADHD symptoms that were previously under control due to lower environmental demands may have caused functional impairment and been brought to clinical attention.^39^

Mental well-being declined globally during the pandemic, especially among children and young adults, with a significant increase in anxiety and depression symptoms.^47,48,49,50^ In Finland, adolescents and young adults reported a significant increase in anxiety and depressive symptoms related to social isolation and insecurity related to the future.^51^ Together, depression and anxiety symptoms may have been mixed with underlying ADHD, with potential overlapping features. Anxiety and depression have, however, been reported to have declined rapidly after the first weeks of the pandemic.^52,53^ Further studies are needed to investigate potential ADHD comorbidities before and during the pandemic time and to determine whether the increasing trend in new ADHD diagnoses or ADHD-related impairment persist in the longer term, even if not induced by pandemic-related environmental stress.

### Strengths and Limitations

This is, to our knowledge, the first population-wide study to assess new ADHD diagnoses and ADHD prevalence before and during the COVID-19 pandemic. The strength of this study is the high coverage of national care and medication registers and thus the ability to capture cases from every service layer in the country, from primary care to hospital units. Diagnostic conventions are harmonized throughout the country.

This study has limitations. A shift in the registry data took place in 2020, when the primary care registry was extended to also cover occupational health care services. Therefore, we may not have full coverage of diagnoses from the private health care sector before 2020. Sensitivity analysis, however, showed that only a fraction of incremental cases came from the occupational health register. Also, obviously, occupational health care is available only for the working-age population in Finland. In addition, medication purchases from the national Medical Reimbursement Register cover prescriptions from private health care and are considered to accurately reflect the presence of ADHD; in clinical practice in Finland, ADHD medications are very rarely used among individuals without ADHD diagnosis. We were not able to comprehensively assess ADHD by subtypes, ie, predominantly inattentive, hyperactive-impulsive, or combined presentations. However, *ICD-10* code F98.8 was included, as it was applied for the inattentive subtype in Finland from 1996 to 2012. According to the Finnish Current Care guidelines of ADHD, *ICD-10* code F90.0 was instructed to be used for all 3 subtypes since 2012,^30^ but the use of the code F98.8 for inattentive-type ADHD may have continued on a small scale afterwards. For tracking the earlier ADHD diagnoses, we relied on the contemporary diagnostic practices in *ICD-9* and *ICD-8*, which may have minor differences compared to *ICD-10*. Unsafe school environment and economic hardship have been shown to increase the risk of ADHD.^54,55,56^ We were unable to assess ethnic or socioeconomic backgrounds of the participants. In general, all Finnish residents have access to public health care services in schools and primary care.

## Conclusions

This nationwide cohort study including the whole Finnish population showed a substantial excessive increase in ADHD diagnoses and ADHD medication use in all age groups and both sexes during the COVID-19 pandemic, with boys and young men being an exception. Our results highlight the unexpected outcomes of exceptional societal conditions on population mental health and demonstrate how living conditions–related stress, such as the pandemic lockdown, may contribute to the incidence of a neurodevelopmental condition with a postulated childhood onset. Further studies are needed to explain the psychological, societal, and biological mechanisms underlying these observations.

## Notes

Supplement 1.
eMethods.

eReferences.
**eFigure 1.** Forest Plot of the Estimated New ADHD Diagnosis Rate Ratios (RR) for 0 to 12 Years, 31 to 55 Years and 56 Years and Older **eFigure 2.** Observed Monthly New Diagnoses of ADHD With Fitted Regression Models **eFigure 3.** Observed Monthly New Diagnoses of ADHD With Fitted Regression Models When Occupational Health–Related New ADHD Diagnoses Were Removed From Data **eFigure 4.** Forest Plot of the Estimated New ADHD Diagnosis Rate Ratios (RR) When New Diagnoses of ADHD From Occupational Health Care Were Removed From Data **eFigure 5.** Rates of New Diagnoses of ADHD, Lifetime Prevalence Rates of ADHD, and Period and Lifetime Prevalence Rates of ADHD Medication Use in the Finnish Population by Age and Sex When Occupational Health–Related New Diagnoses of ADHD Were Removed From Data

Supplement 2. **eTable 1.** Study Cohort Characteristics **eTable 2.** Estimates of the Model Covariates for Each Group From Poisson or Negative Binomial Regression Model **eTable 3.** The Number of Occupational Health Related New Diagnoses of ADHD and the Proportion From all New Diagnoses of ADHD Between 2020 and 2022 **eTable 4.** Excessive New Diagnoses of ADHD Related to COVID-19 Pandemic in Finland When Occupational Health–Related Observations are Removed **eTable 5.** Estimates of the Model Covariates for Each Group From Poisson or Negative Binomial Regression Model When Occupational Health–Related Observations are Removed **eTable 6.** New Diagnoses of ADHD and Period Prevalence of ADHD Medication Purchase Measured at 3 Time Periods When Occupational Health–Related Observations are Removed **eTable 7.** Lifetime Prevalence of ADHD and ADHD Medicine Purchase Measured at 3 Time Points When Occupational Health–Related Observations are Removed

Supplement 3.
Data Sharing Statement
