# Supplementary material for: Attention-Deficit/Hyperactivity Disorder Diagnoses in Finland During the COVID-19 Pandemic
Source: JAMA Netw Open. 2024 Jun 27;7(6):e2418204. doi: 10.1001/jamanetworkopen.2024.18204 (PMC11211961; doi:10.1001/jamanetworkopen.2024.18204)
Supplement: Supplement 1. — eMethods. eReferences. eFigure 1. Forest Plot of the Estimated New ADHD Diagnosis Rate Ratios (RR) for 0 to 12 Years, 31 to 55 Years and 56 Years and Older eFigure 2. Observed Monthly New Diagnoses of ADHD With Fitted Regression Models eFigure 3. Observed Monthly New Diagnoses of ADHD With Fitted Regression Models When Occupational Health–Related New ADHD Diagnoses Were Removed From Data eFigure 4. Forest Plot of the Estimated New ADHD Diagnosis Rate Ratios (RR) When New Diagnoses of ADHD From Occupational Health Care Were Removed From Data eFigure 5. Rates of New Diagnoses of ADHD, Lifetime Prevalence Rates of ADHD, and Period and Lifetime Prevalence Rates of ADHD Medication Use in the Finnish Population by Age and Sex When Occupational Health–Related New Diagnoses of ADHD Were Removed From Data [file jamanetwopen-e2418204-s001.pdf]

Supplemental Online Content

Auro K, Holopainen I, Perola M, Havulinna AS, Raevuori A. Excessive attention-deficit/hyperactivity disorder diagnoses in Finland during the COVID-19 pandemic. *JAMA Netw Open*. 2024;7(6):e2418204. doi:10.1001/jamanetworkopen.2024.18204

eMethods.

eReferences.

eFigure 1. Forest Plot of the Estimated New ADHD Diagnosis Rate Ratios (RR) for 0 to 12 Years, 31 to 55 Years, and 56 Years and Older

eFigure 2. Observed Monthly New Diagnoses of ADHD With Fitted Regression Models

eFigure 3. Observed Monthly New Diagnoses of ADHD With Fitted Regression Models When Occupational Health–Related New ADHD Diagnoses Were Removed From Data

eFigure 4. Forest Plot of the Estimated New ADHD Diagnosis Rate Ratios (RR) When New Diagnoses of ADHD From Occupational Health Care Were Removed From Data

eFigure 5. Rates of New Diagnoses of ADHD, Lifetime Prevalence Rates of ADHD, and Period and Lifetime Prevalence Rates of ADHD Medication Use in the Finnish Population by Age and Sex When Occupational Health–Related New Diagnoses of ADHD Were Removed From Data

This supplemental material has been provided by the authors to give readers additional information about their work.

© 2024 Auro K et al. *JAMA Network Open.*

eMethods

ADHD case definition

For each person, ADHD was defined with the first appearance of ADHD diagnosis (ICD-10: F90.0, F90.1, F90.8, F90.9, F98.8; ICD-9: 3140; ICD-8: 308.99, 308.3 and 309) in the Care Register for Health Care (HILMO) or Primary Care Outpatient Register (AVOHILMO) or as the first ADHD medication purchase (ATC: N06BA09, N06BA04, N06BA02 and N06BA12) from the Prescription Medicine Purchase Register. HILMO includes data from hospital inpatient and outpatient clinics between 1969 and June 30th, 2022, and AVOHILMO from primary care since 2011. The prescription medicine purchase register covered data from 1995 to June 30th, 2022. AVOHILMO started to cover occupational health care in 2019, and this might have affected the monthly trends of ADHD incidence. In sensitivity analyses we studied if the addition of occupational health care diagnoses had affected the observed new diagnoses of ADHD during pandemic period by filtering out the occupational health related new diagnoses of ADHD. Occupational health related new diagnoses of ADHD were defined as the first observed ADHD diagnosis that was given in occupational health care as documented in AVOHILMO, without any following non-occupational related ADHD diagnosis from HILMO or AVOHILMO or medication purchase within a year of follow-up.

Main outcome: new ADHD diagnoses

The new diagnoses of ADHD were analyzed in three different ways and these analyses were carried independently for each age and sex specific groups. First, we modelled the monthly rates for new diagnoses of ADHD between January 2015 to June 2022. Second, we computed the rate of new diagnoses of ADHD, expressed as percentages, using half-year intervals covering January 1st, 2015, to June 30th, 2022. Third, we computed the number of new diagnoses of ADHD per 100,000 for three different time periods: first one covered year 2015, second from April 1, 2019 to March 31, 2020, and third from July 1, 2021 to June 30, 2022. The new diagnoses rate was computed as the number of people with new diagnosis of ADHD within observed time window divided by the population size of the group which was estimated at the mid-point of the observed time window. The uncertainty related to the estimated population size was estimated with confidence intervals based on Poisson distribution. The uncertainty of the estimated rates and proportions were estimated with Clopper-Pearson confidence intervals[1]:

$$\left[B\left(\frac{\alpha}{2};x;n-x+1\right),B\left(1-\frac{\alpha}{2};x+1;n-x\right)\right] \tag{1}$$

where α is the set confidence level and x is the number of successes from n trials. $B(x;\alpha;\beta)$ is the quantile function of the beta distribution in which x is the set confidence level and α and β are the shape parameters of the beta distribution.

The possible influence of the COVID-19 pandemic on the observed new diagnoses of ADHD was modelled with multivariable Poisson or negative binomial regression which was done separately for each group defined by age and sex. Model selection was based on minimizing the Akaike information criterion (AIC). Poisson model was used with >55 y women and men and negative binomial model was used with remaining groups. In the selected regression model, the dependent variable was monthly observed new diagnoses of ADHD and independent variables accounted for the overall increasing trend, seasonality, and possible effect of the COVID-19 pandemic in observed new diagnoses of ADHD. The overall increasing trend of new diagnoses of ADHD was adjusted in the models with continuous day count, that was defined as number of days between the start of each observed month to the baseline, January 1st 2015. This variable was rescaled in results to present the yearly effect (*Year*) by multiplying with approximate number of days in a year, 365.24. There seemed to be monthly variation in the number of observed new diagnoses of ADHD: especially among younger population there was a drop in number of new diagnoses during summer months and December. This seasonal effect was adjusted in the regression models with categorical variable describing the effect of each month on observed new diagnoses of ADHD, with *January* used as the baseline. A dummy indicator variable was used to model the possible increase in the observed number of new diagnoses of ADHD after the start of the COVID-19 pandemic: the variable was set to 0 before the onset of the pandemic and 1 after the start of the pandemic, defined as April 1st, 2020. The number of individuals at risk were estimated as mid-month population count for each group.

The parameter estimates of each regression model and their 95% confidence intervals and p-values based on two-tailed Z test are presented in eTable 2 and in eTable 5. The estimate of the Pandemic period variable was positive and statistically significant in 0-12 and 13-20 years old girls, 21-30 and 31-55 years old women and in 21-30 and 31-

© 2024 Auro K et al. *JAMA Network Open.*

55 years old men which can be seen also in the estimated rate ratio of new ADHD diagnoses between pandemic period vs. before pandemic (Figure 2, eFigure 1 and eFigure 5). The estimate of the variable Year was positive and statistically significant in all regression models. In the seasonal variable, summer months from *June* to *August* were negative and statistically significant in age groups of 0-12, 13-20 and 21-30 years old. The estimate of *July* was negative and statistically significant in all age groups except among 56 and over women. Similarly, the estimate of *December* was negative and statistically significant in age groups of 0-12, 13-20, 21-30 and 31-55 years old. In other words, these months in listed groups had statistically significantly smaller number of new ADHD diagnoses compared to *January*, the baseline month. These results indicated that even though there was an increasing trend of new ADHD diagnoses with potential seasonal fluctuations, the increase in the number of new ADHD diagnoses on top of the existing trend after the start of the COVID-19 pandemic was significant in 0-12 and 13-20 years old girls, 21-30 and 31-55 years old women, and in 21-30 and 31-55 years old men. The observed new diagnoses of ADHD per month and the predicted values of the regression model with their normal approximated 95% confidence intervals are presented in eFigure 2 and eFigure 3 for each group. The fitted regression models well explain the observed new diagnoses of ADHD and possible trend change in new ADHD diagnoses after the start of the pandemic.

Additional new diagnoses of ADHD during the COVID-19 pandemic

Additional new diagnoses of ADHD were estimated for each group over the pandemic period by computing the difference between the predicted values of the fitted regression model where the pandemic effect was included and excluded, i.e., the variable representing the potential effect of the COVID-19 pandemic was set to 1 and to 0 over the observed months that were considered to be within the pandemic period, i.e. from April 2020 to June 2022. The sum over this difference presents the estimate of overall additional cases during the pandemic period. The 95% confidence intervals for the additional cases were computed with bootstrapping by sampling 10000 samples from the model distributions with the predicted values and applying the transformation on the sampled random variables. The estimated additional values are purely based on the regression coefficient estimates and on the assumptions regarding the Poisson and negative binomial regression models. These estimates do not take into account the uncertainty in the regression coefficient estimates. The additional new diagnoses of ADHD are presented in Table 2 and in eTable 4. These tables present the observed number of new ADHD diagnoses over the pandemic period, the number of new ADHD diagnoses that are considered as additional from this period, and the proportion of the additional cases from observed including the bootstrapped 95% confidence intervals.

Secondary outcome: lifetime prevalence for ADHD and ADHD medication use and period prevalence for ADHD medication use

Lifetime prevalence was defined at certain time points as the number of prevalent people divided by the population size at the observed time point. Prevalent ADHD was defined as person having either an existing ADHD diagnosis or at least one ADHD medication purchase before the prevalence time point Figure 1 presents the lifetime prevalence for ADHD and ADHD medication use as percentages that is defined on every half-year point, starting at January 2015, and ending to June 2022. Table 2 and eTable 7 present the lifetime prevalence for ADHD and ADHD medication use that was computed for three different time points: December 31st, 2015, March 31st, 2020, and June 30th, 2022. Clopper-Pearson confidence intervals from Equation 1 were used with the prevalence rates.

Also, period prevalence for ADHD medication use was computed for three different time periods and half-year intervals, similarly to the new diagnoses of ADHD presented in Table 1 and eTable 6. Here, prevalent ADHD medication use was defined as person having at least one ADHD medication purchase within the observed time interval. The period prevalence was computed as the proportion of the number of people with prevalent ADHD medication use from the population size that was estimated with the mid-point population of the observed interval. The confidence intervals for population size were based on Poisson distribution, and Clopper-Pearson confidence intervals from Equation 1 were used for period prevalence rate.

eReferences

1. Clopper CP, Pearson ES. *The Use of Confidence or Fiducial Limits Illustrated in the Case of the Binomial*. Biometrika. 1934;26(4):404—413.

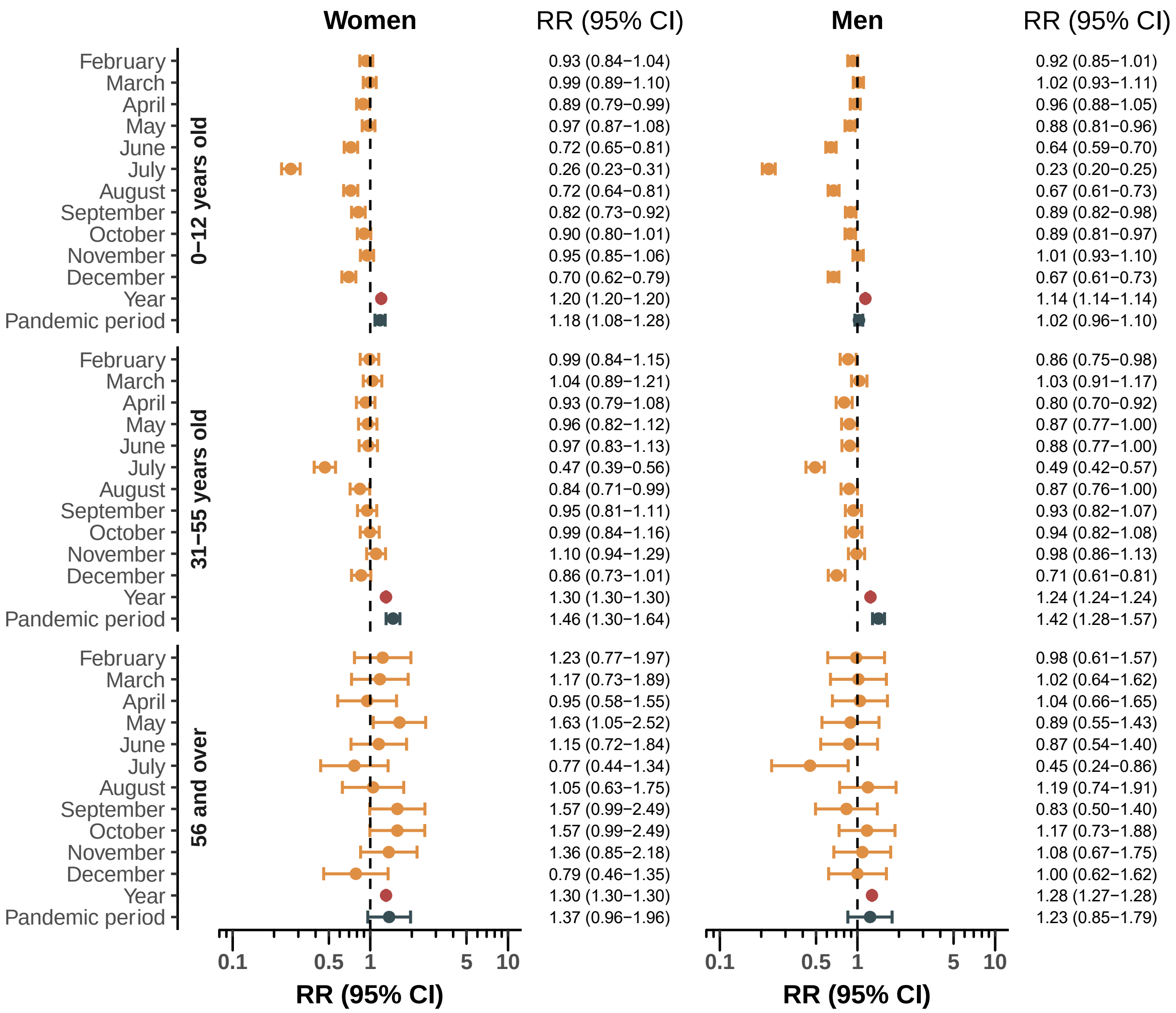

eFigure 1. Forest plot of the estimated new ADHD diagnose rate ratios (RR) for 0 to 12 years, 31-55 years, and 56 and older.

RR and their 95% confidence intervals were obtained from the multivariable Poisson or negative binomial regression that was modeled separately for women and men in specified age groups. This figure presents the results for 0-12 years old (first row), 31-55 years old (second row) and 56 and over (third row). Women are presented on the left side of the figure and men are presented on the right. Numerical values listed in the figure are rounded to two decimal places. Poisson regression was used with 56 and over women and men and negative binomial regression was used with rest of the groups. The models were adjusted for overall increasing trend that was modeled with continuous day count, here rescaled to present yearly effect (red), seasonal effect as categorical variable for each month (orange), in which January was used as baseline, and possible effect of the COVID-19 pandemic with dummy variable (black). The dots present the estimated RR that is exponential transformation of the parameter estimate and horizontal bars present their 95% z confidence intervals, and they are colored according to model variables. The RR of the pandemic period (black) describes the ratio of the new ADHD diagnose rates during vs. before pandemic in each model.

© 2024 Auro K et al. *JAMA Network Open.*

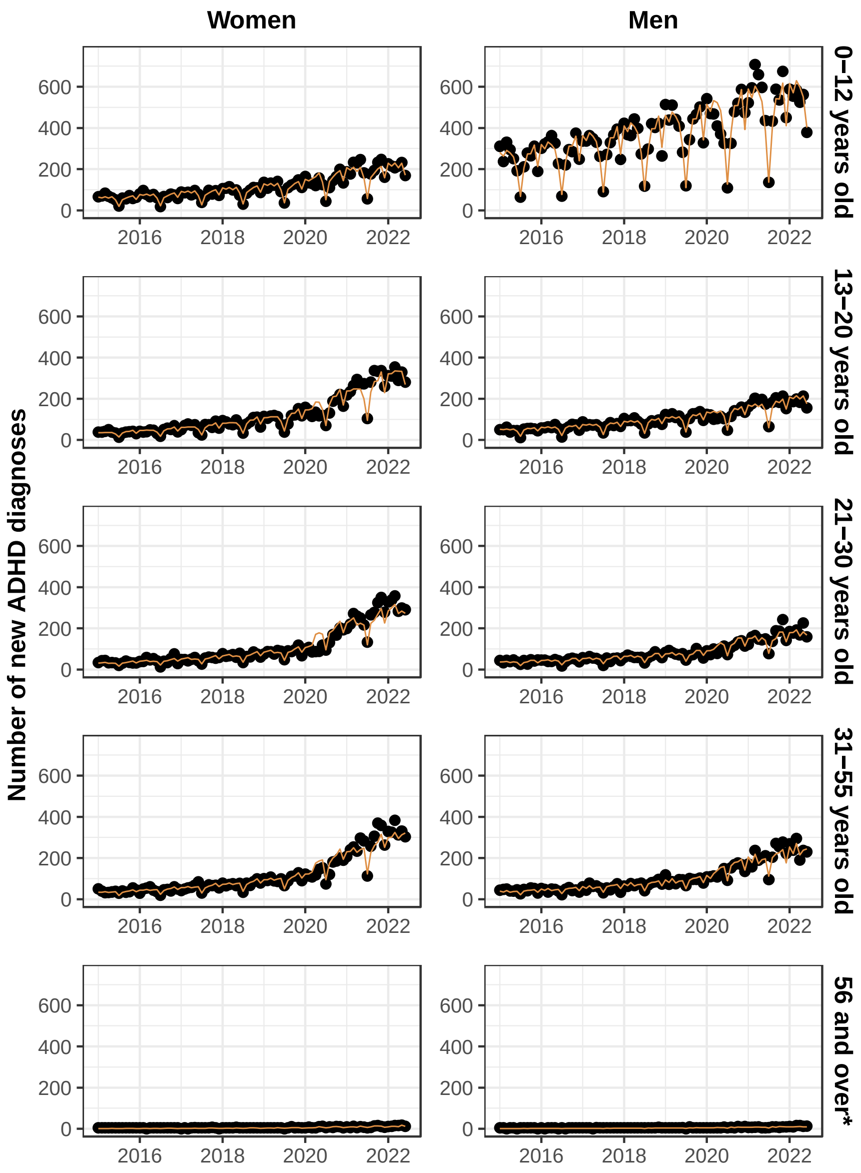

*Poisson model was used in stead of negative binomial model.

eFigure 2. Observed monthly new diagnoses of ADHD with fitted regression models.

The observed monthly new ADHD diagnoses (black dots) from January 2015 to June 2022 were modelled with Poisson or negative binomial regression for each group age and sex specific group separately. The plots show predicted values of the fitted regression models (orange line) with their normal approximated 95% confidence intervals (orange colored area). The start of the COVID-19 pandemic in Finland, April 2020, is marked with dashed vertical line. Observed values that are between 1 and 5 are rounded to 5.

© 2024 Auro K et al. *JAMA Network Open.*

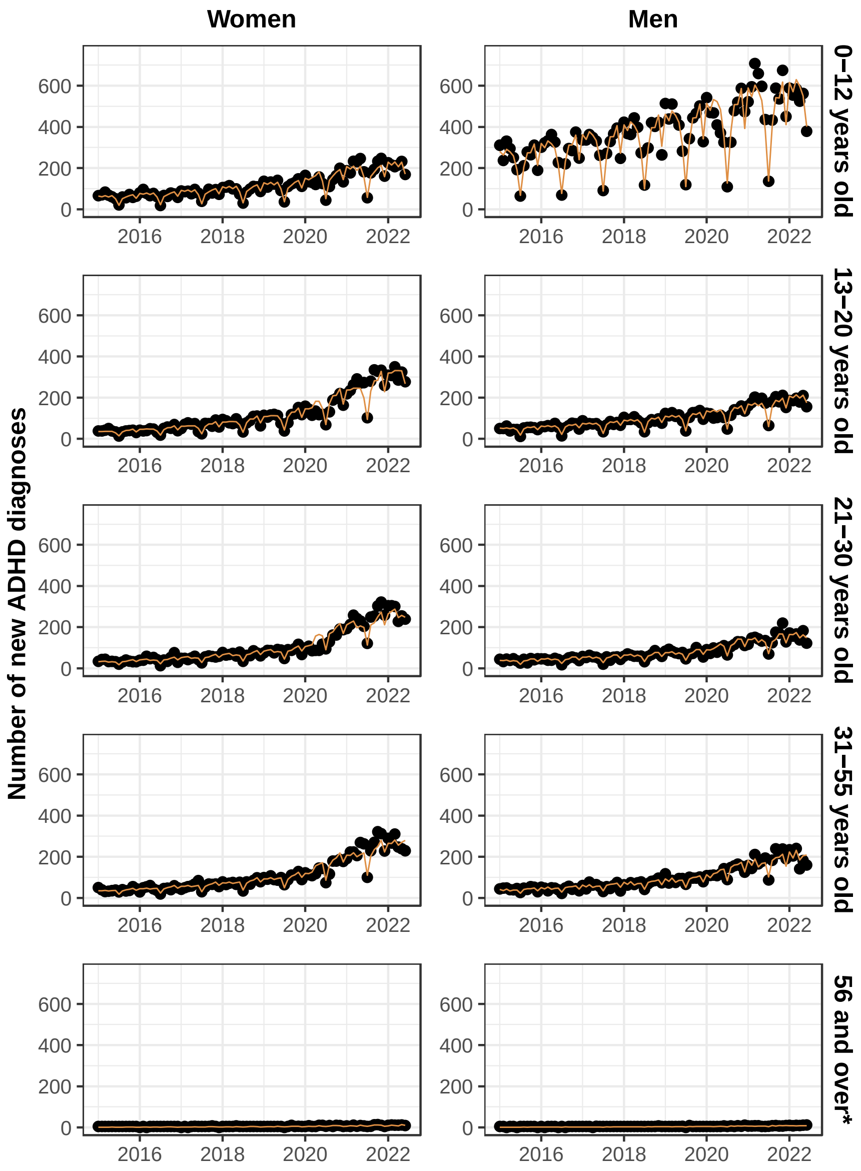

*Poisson model was used in stead of negative binomial model.

eFigure 3. Observed monthly new diagnoses of ADHD with fitted regression models when occupational health-related new ADHD diagnoses were removed from data.
The observed monthly new ADHD diagnoses (black dots) from January 2015 to June 2022 were modelled with Poisson or negative binomial regression for each age and sex specific group separately. The plots show predicted values of the fitted regression models (orange line) with their normal approximated 95% confidence intervals (orange colored area). The start of the COVID-19 pandemic in Finland, April 2020, is marked with dashed vertical line. Observed values that are between 1 and 5 are rounded to 5.

© 2024 Auro K et al. *JAMA Network Open.*

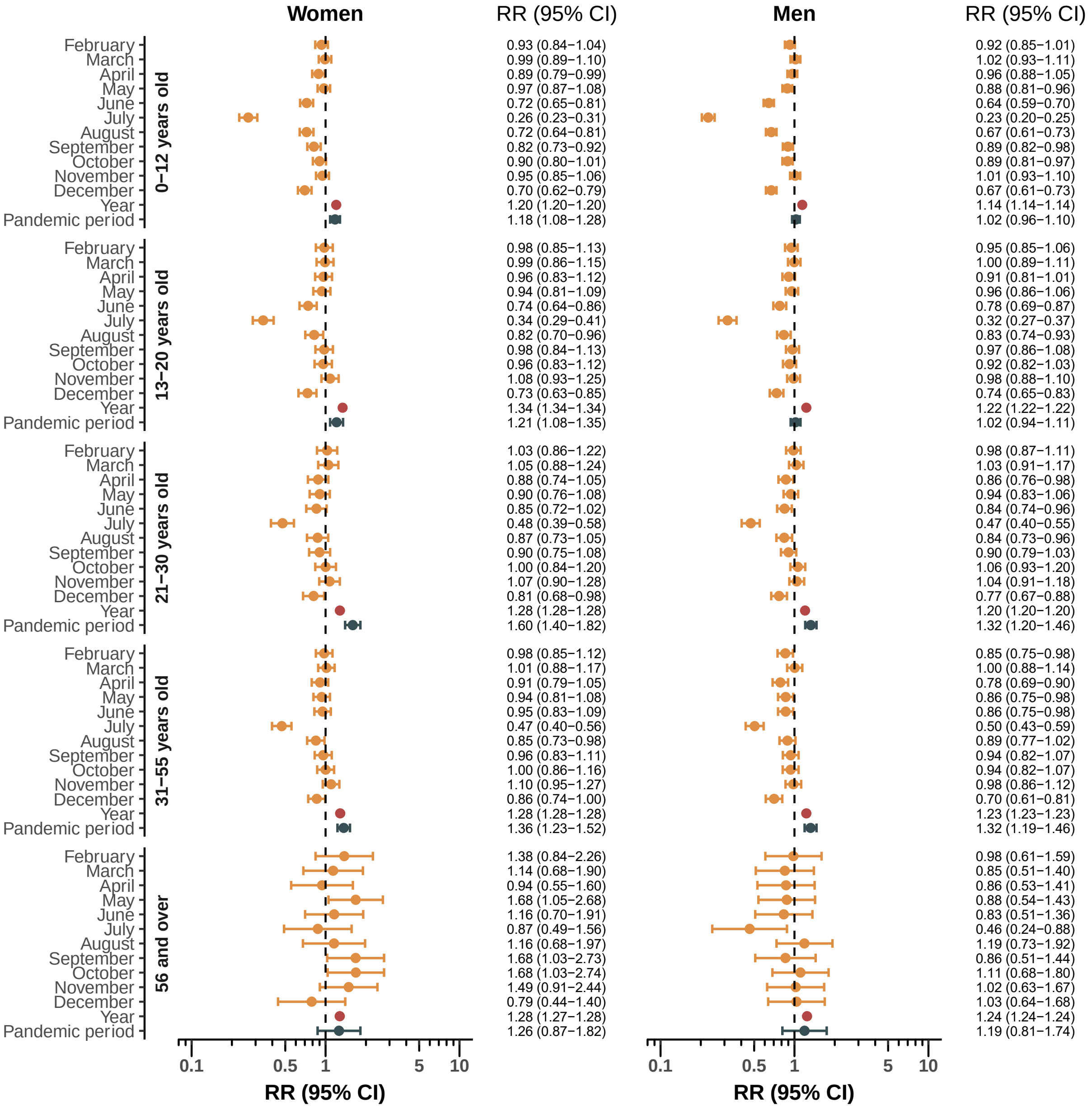

eFigure 4. Forest plot of the estimated new ADHD diagnose rate ratios (RR) when new diagnoses of ADHD from occupational health care were removed from data.

RR and their 95% confidence intervals were obtained from the multivariable Poisson or negative binomial regression that was modeled separately for women and men in specified age groups. Age groups were divided in following way: 0-12 years old (first row), 13-20 years old (second row), 21-30 years old (third row), 31-55 years old (fourth row) and 56 and over (fifth row). Women are presented on the left side of the figure and men are presented on the right. Numerical values listed in the figure are rounded to two decimal places. Poisson regression was used with 56 and over women and men and negative binomial regression was used with rest of the groups. The models were adjusted for overall increasing trend that was modeled with continuous day count, here rescaled to present yearly effect (red), seasonal effect as categorical variable for each month (orange), in which January was used as baseline, and possible effect of the COVID-19 pandemic with dummy variable (black). The dots present the estimated RR that is exponential transformation of the parameter estimate and horizontal bars present their 95% z confidence intervals, and they are colored according to model variables. The RR of the pandemic period (black) describes the ratio of the new ADHD diagnose rates during vs. before pandemic in each model.

© 2024 Auro K et al. *JAMA Network Open.*

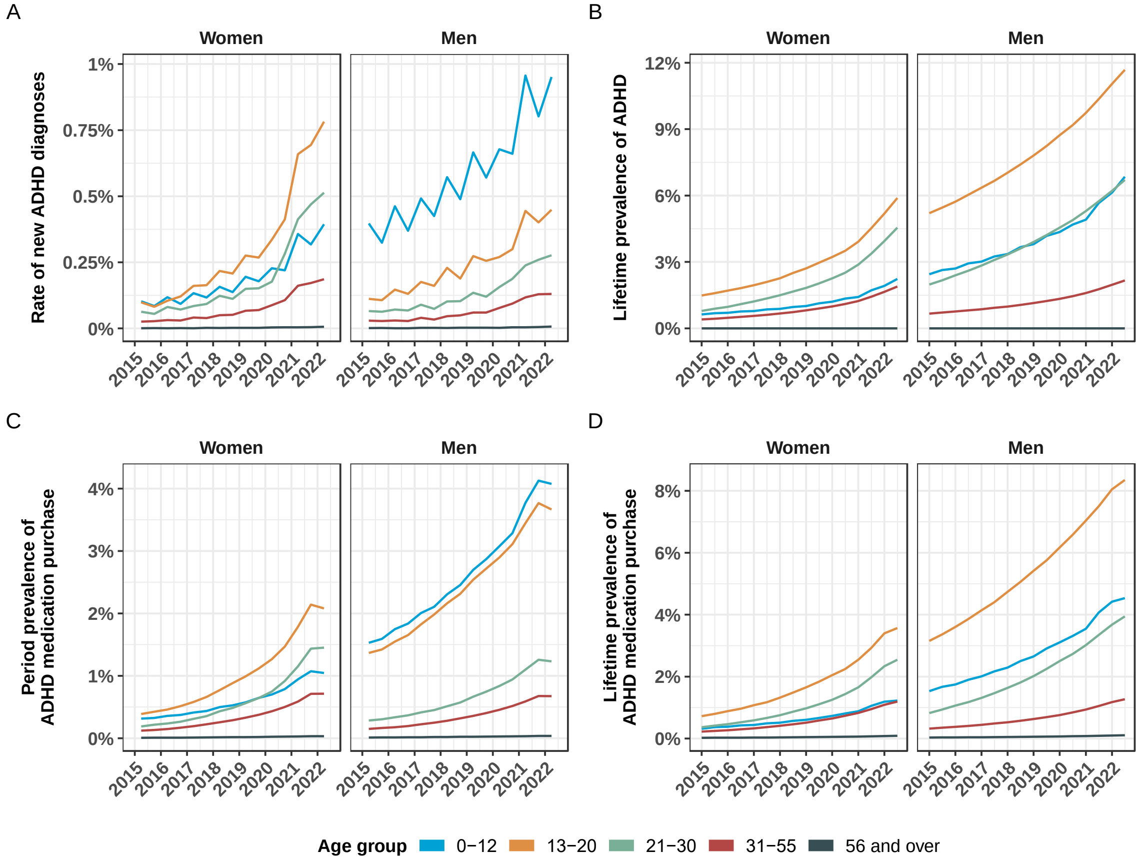

eFigure 5. Rates of new diagnoses of ADHD, lifetime prevalence rates of ADHD and period and lifetime prevalence rates of ADHD medication use in the Finnish population by age and sex when occupational health related new diagnoses of ADHD were removed from data.

Rates of the new diagnoses of ADHD and period ADHD prevalence were computed with half-year intervals, and lifetime prevalence was estimated every half-year. The population at risk was estimated with mid-point population for new diagnoses and period prevalence. Age groups were divided in following way: 0-12 (blue), 13-20 (orange), 21-30 (green), 31-55 (red), 56 and over (black). Colored area around the estimated rate represents the Clopper-Pearson 95% confidence interval. The dashed vertical line marks the beginning of the pandemic in Finland (April 1st, 2020). (A) New diagnosis rate of ADHD (%); (B) Lifetime prevalence of ADHD (%); (C) Period prevalence (%) of individuals with any ADHD medication purchase within the half-year interval; (D) Lifetime prevalence of ADHD medication purchases (%).

© 2024 Auro K et al. *JAMA Network Open.*
