# Supplementary material for: Attention-Deficit/Hyperactivity Disorder Diagnoses in Finland During the COVID-19 Pandemic
Source: JAMA Netw Open. 2024 Jun 27;7(6):e2418204. doi: 10.1001/jamanetworkopen.2024.18204 (PMC11211961; doi:10.1001/jamanetworkopen.2024.18204)
Supplement: Supplement 3. — Data Sharing Statement [file jamanetwopen-e2418204-s003.pdf]

Data Sharing Statement

Auro. Excessive Attention-Deficit/Hyperactivity Disorder Diagnoses in Finland During the COVID-19 Pandemic. *JAMA Netw Open*. Published June 27, 2024. doi:10.1001/jamanetworkopen.2024.18204

Data
Data available: No

Additional Information
Explanation for why data not available: Individual patient data cannot be shared according to the patient consent
